# AZD1480 delays tumor growth in a melanoma model while enhancing the suppressive activity of myeloid-derived suppressor cells

**DOI:** 10.18632/oncotarget.2254

**Published:** 2014-07-25

**Authors:** Sarah K. Maenhout, Stephanie Du Four, Jurgen Corthals, Bart Neyns, Kris Thielemans, Joeri L. Aerts

**Affiliations:** ^1^ Laboratory of Molecular and Cellular Therapy, Department of Immunology-Physiology, Vrije Universiteit Brussel, Brussels, Belgium; ^2^ Department of Medical Oncology, Universiteit Ziekenhuis Brussel, Brussels, Belgium

**Keywords:** signal transducer and activator of transcription 3, JAK1/2 inhibitors, tumor immunology, myeloid-derived suppressor cells, immunosuppression

## Abstract

AZD1480 is a potent, competitive small-molecule inhibitor of JAK1/2 kinase which inhibits STAT3 phosphorylation and tumor growth. Here we investigated the effects of AZD1480 on the function of different immune cell populations in a melanoma model. When MO4 tumor-bearing mice were treated with AZD1480 we observed a strong inhibition of tumor growth as well as a prolonged survival. Moreover, a significant decrease in the percentage of myeloid-derived suppressor cells (MDSCs) was observed after treatment with AZD1480. However, AZD1480 enhanced the suppressive capacity of murine MDSCs while at the same time impairing the proliferative as well as the IFN-γ secretion capacity of murine T cells. The addition of AZD1480 to co-cultures of human MDSCs and T cells does not affect the suppressive activity of MDSCs but it does reduce the IFN-γ secretion and the proliferative capacity of T cells. We showed that although AZD1480 has the ability to delay the tumor growth of MO4 tumor-bearing mice, this drug has detrimental effects on several aspects of the immune system. These data indicate that systemic targeting of the JAK/STAT pathway by JAK1/2 inhibition can have divergent effects on tumor growth and anti-tumor immune responses.

## INTRODUCTION

Conventional chemotherapeutic agents used for the treatment of cancer patients act by exerting toxic effects on all dividing cells, leading to severe damage of normal tissue which results in side effects including but not limited to myelosuppression, alopecia and gastrointestinal problems. Therefore more specific therapies targeting key elements that play a role in tumor initiation and progression are being developed so as to specifically target malignant cells with severely reduced side-effects[1,2]. There are over 20 small-molecule protein kinase inhibitors currently approved or under investigation for the treatment of human diseases. Inhibitors of protein kinases continue to be the focus of drug discovery programs, especially in the field of oncology[3,4].

The link between inflammation and cancer has been well established. Under normal physiological conditions, inflammation is a self-limiting process, but dysfunctions in one of the inflammatory pathways can lead to pathogenesis and eventually to tumorigenesis[5]. The intracellular signalling pathways responsible for the inflammatory process are very complex, implicating dozens of molecules involved in several different signalling pathways. However, it has become clear that signal transducer and activator of transcription 3 (STAT3) is a kind of master switch that is connected to most signalling pathways related to both oncogenesis and tumor-associated inflammation[6,7]. Activation of STAT3 has been described in nearly 70% of solid and hematological tumors, emphasizing the importance of STAT3 as a direct target in cancer therapy[8,9]. Activated tyrosine kinases phosphorylate STAT3 (P-STAT3), which in turn forms dimers that translocate to the nucleus, where they directly regulate gene expression. In addition to upregulating numerous genes involved in proliferation, survival, invasion and metastasis of tumor cells, STAT3 induces the expression of many cytokines, such as IL-6, IL-10, TGF-β and VEGF, that are associated with cancer-promoting inflammation[10,11]. Moreover, persistent activation of STAT3 is not limited to the tumor itself: it is also transmitted to stromal inflammatory cells in the tumor microenvironment. STAT3 signalling in innate immune cells is required for the immunosuppressive and tumor-promoting effects of myeloid-derived suppressor cells (MDSCs) and tumor-associated macrophages (TAMs)[12–14]. STAT3 also mediates regulatory T cell (T_reg_) expansion in tumors and is necessary for the development of Th17 cells[15], which can promote tumor growth. Because STAT3 induces the expression of cytokines, growth factors and angiogenic factors, and the associated receptors in turn activate STAT3, a feedforward loop is established between tumor cells and immune cells. As a consequence, persistent activation of STAT3 mediates both the propagation of tumor-promoting inflammation and the suppression of anti-tumor immunity, thus forming a promising target to improve cancer therapy by modulating immune responses[16].

Current attempts to target the JAK-STAT3 pathway include the use of IL-6 and IL-6 receptor blocking antibodies[17], the use of specific STAT3 inhibitors[18–20] and JAK inhibitors[21]. AZD1480 is a potent, competitive small-molecule inhibitor of JAK1/2 kinases, that is capable of blocking STAT3 phosphorylation and inhibiting tumor growth in a STAT3-dependent manner[22]. In several different murine and human tumor cell lines, including 786-O (renal cell carcinoma), DU145 (prostate cancer), MDAH2774 (ovarian carcinoma), MDA-MB-468 (breast cancer), Renca cells (renal carcinoma) and several human myeloma cell lines, it has been shown that AZD1480 is able to downregulate P-STAT3 expression in tumor cells resulting in a delayed tumor growth *in vivo*[22–24]. However, none of these studies looked at the direct effects of AZD1480 on the immune system. Therefore, in the present study we investigated the effects of AZD1480 on the phenotype and function of different immune cell populations present in the tumor microenvironment, including T cells, dendritic cells (DCs) and MDSCs, in a murine melanoma model and on peripheral blood mononuclear cells obtained from melanoma patients. We show that, although AZD1480 has the ability to delay tumor growth and prolong survival of MO4-tumor bearing mice, the inhibition of JAK1/2 has detrimental effects on several aspects of the immune system of these mice.

## RESULTS

### AZD1480 inhibits P-STAT3 expression but does not induce apoptosis in murine melanoma cell lines *in vitro*

**Figure 1:**
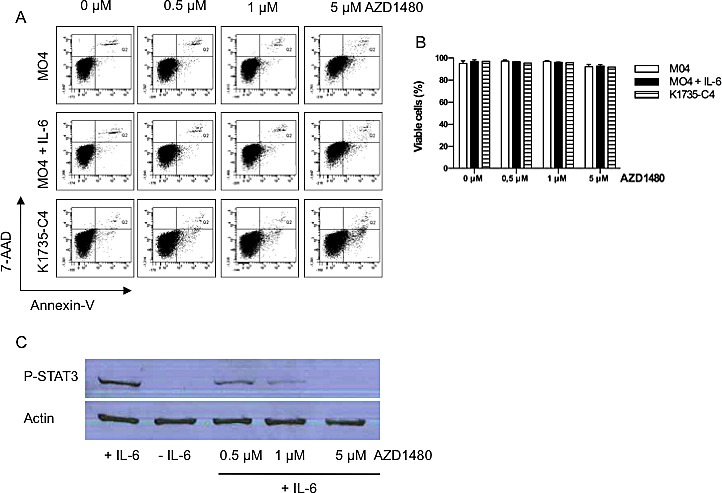
AZD1480 inhibits P-STAT3 expression but does not induce apoptosis in murine melanoma cell lines *in vitro* MO4 cells, MO4 cells pretreated with IL-6 (50 ng/ml) and K1735-C4 melanoma cells were treated with the indicated doses of AZD1480 and after 48 hours the percentage of apoptotic cells was determined by flow cytometry using Annexin-V/7-AAD staining. A. Represenative FACS profile of Annexin-V/7-AAD staining of the different cell lines treated with AZD1480. B. Overview of the percentage of viable cells (defined as Annexin-V^−^/7-AAD^−^ cells) in different mouse melanoma cell lines 48 hours after the addition of different concentrations of AZD1480. Results of 3 independent experiments are shown as mean ± SEM. C. MO4 cells pretreated with IL-6 (50 ng/ml) were treated with the indicated doses of AZD1480 and after 6 hours the level of P-STAT3 expression was determined by western blot. One representative blot of 2 indepenent experiments is shown.

Since it is well known that inhibition of STAT3 signalling can induce apoptosis in tumor cells[25] we determined the apoptotic effects of AZD1480 in different murine melanoma cell lines *in vitro*. The MO4 cell line, which lacks constitutive expression of P-STAT3, was treated with IL-6, in order to induce P-STAT3 expression and the K1735-C4 cell line, characerized by constitutive expression of P-STAT3, was treated with different doses of AZD1480 for 48 hours. Even at the highest concentration we tested (5 μM), AZD1480 failed to induce apoptosis in all tested cell lines as evaluated by Annexin-V/7-AAD staining (Figure 1A,B). However, a dose-dependent downregulation of P-STAT3 expression in MO4 melanoma cells was apparant 6 hours after the treatment with AZD1480. At a concentration of 5 μM, AZD1480 completely abrogated the P-STAT3 expression in IL-6 treated MO4 cells (Figure 1C). Comparable effects were seen in a human melanoma cell line (Supplementary Figure 1) These data demonstrate that AZD1480 is able to inhibit induced activation of P-STAT3 in the MO4 cell line, without affecting the viability of these cells.

### AZD1480 inhibits the growth of subcutaneously implanted MO4 melanoma tumors and prolongs survival of tumor-bearing mice by inhibiting P-STAT3 expression within the tumor environment

**Figure 2:**
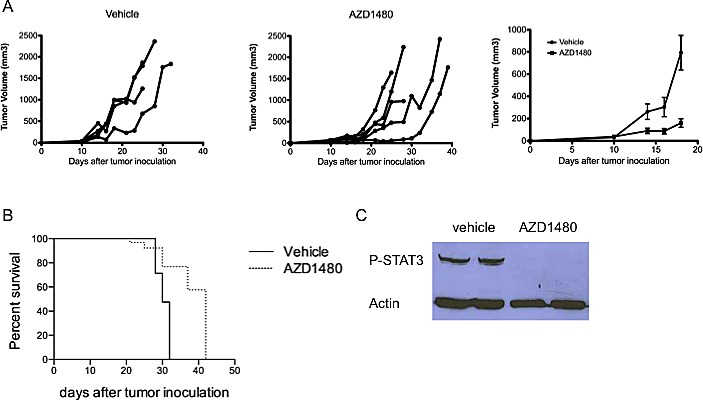
AZD1480 inhibits the growth of subcutaneously implanted MO4 melanoma tumors and prolongs survival of tumor-bearing mice by inhibiting P-STAT3 expression within the tumor environment MO4 tumor-bearing mice were treated with AZD1480 at 30 mg/kg or vehicle control by oral gavage bid for 7 days. A. Individual growth curves of melanoma tumor-bearing mice treated with vehicle control (left panel) or AZD1480 (middle panel). Mean tumor volume of mice treated with vehicle control or AZD1480 is shown in the right panel. One representative of 2 independent experiments with each time 5 mice per group is shown. B. Survival curve of MO4 tumor-bearing mice treated with vehicle control or AZD1480. One representative of 2 independent experiments with each time 5 mice per group is shown. C. Two mice of each treatment group were sacrificed 2 hours after the last dosing and whole-cell lysates were prepared and subjected to western blot analysis for the expression of P-STAT3. One representative blot of 2 independent experiments is shown.

We next wanted to investigate the *in vivo* anti-tumor effects of AZD1480 in a murine melanoma model. MO4 cells were subcutaneously injected in the flank of C57BL/6 mice and when tumors were palpable AZD1480 treatment was initiated. Mice were treated with AZD1480 at 30 mg/kg or with vehicle by oral gavage twice a day for 7 days. We observed a strong inhibition of tumor growth in AZD1480-treated mice compared with the vehicle-treated group (Figure 2A), as well as a prolonged survival of AZD1480-treated mice compared to the vehicle control group (median survival of 42 *versus* 30 days, respectively; Figure 2B). Western blot analysis of whole tumor lysates, obtained two hours after the last dosing of AZD1480 or vehicle, showed a complete inhibition of P-STAT3 expression by AZD1480 treatment (Figure 2C). These results indicate that AZD1480 has potent antitumor effects *in vivo* in this melanoma model, which is associated with inhibition of STAT3 signalling in the tumor microenvironment.

### *In vivo* AZD1480 treatment induces profound changes in the immune cell composition in both the spleen and the tumor microenvironment

**Figure 3:**
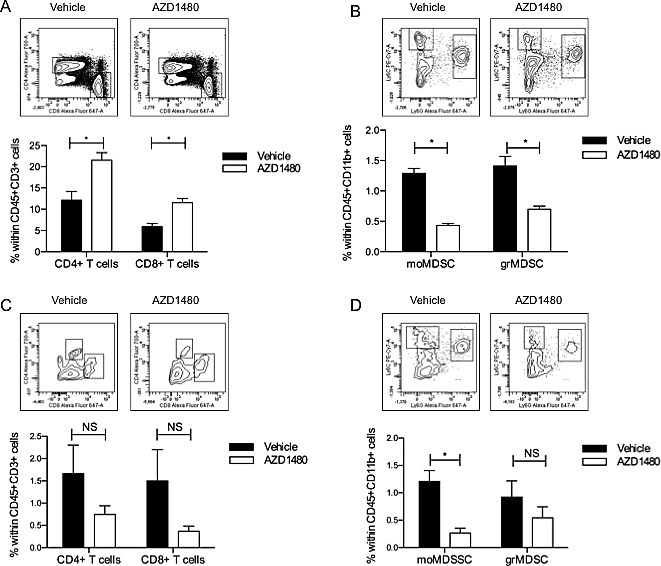
*In vivo* AZD1480 treatment induces profound changes in the immune cell compostion in both the spleen and the tumor microenvironment MO4 tumor-bearing mice were treated with AZD1480 at 30 mg/kg or vehicle control by oral gavage twice a day for 7 days. Two hours after the last dosing mice were sacrificed and single cell suspensions of spleen and tumor were made. Different immune cell populations were subsequently analysed by flow cytometry. A. Percentage of CD45^+^CD3^+^ T cells in the spleen of treated mice. Within the CD45^+^CD3^+^ T cells the percentage of CD4^+^ and CD8^+^ T cells was determined. B. Percentage of myeloid cells (defined as CD45^+^CD11b^+^ cells) in the spleen of treated mice. Within this population the percentage of the different subsets of myeloid-derived suppressor cells (moMDSC: CD45^+^CD11b^+^Ly6C^+^Ly6G^−^ and grMDSC: CD45^+^CD11b^+^Ly6G^+^Ly6C^int^) was determined. C. Percentage of CD45^+^CD3^+^ T cells in the tumor of treated mice. Within the CD45^+^CD3^+^ T cells the percentage of CD4^+^ and CD8^+^ T cells was determined. D. Percentage of myeloid cells (defined as CD45^+^CD11b^+^ cells) in the tumor of treated mice. Within this population the percentage of the different subsets of myeloid-derived suppressor cells (moMDSC: CD45^+^CD11b^+^Ly6C^+^Ly6G^−^ and grMDSC: CD45^+^CD11b^+^Ly6G^+^Ly6C^int^) was determined. Three independent experiments were performed (with each time 3 mice per group) and results are presented as mean ± SEM.

The tumor microenvironment is composed of a complex network of immune cells, which can either inhibit or promote tumor growth. Since we observed a significant anti-tumor effect of AZD1480 we wondered whether AZD1480 influences the immune cell composition in the spleen and within the tumor microenvironment. In the spleen of AZD1480 treated mice we observed a significant increase in the percentages of both CD4^+^ and CD8^+^ T cells compared to vehicle control treated mice (Figure 3A). While we did not observe differences in the percentage of dendritic cells (DCs), nor in the maturation status of these cells (data not shown), we did observe a significant decrease in the percentage of both monocytic MDSCs (moMDSC; CD11b^+^Ly6C^+^Ly6G^−^) and granulocytic MDSCs (grMDSC; CD11b^+^Ly6C^low^Ly6G^+^; Figure 3B) after treatment with AZD1480. In contrast, within the tumor microenvironment, we observed a significant decrease in the percentage of CD45^+^ cells (data not shown) when mice were treated with AZD1480. Within the CD45^+^ cell population we evaluated the presence of T cells, DCs and MDSCs. The percentage of both tumor-infiltrating CD4^+^ and CD8^+^ T cells was dramatically decreased in AZD1480 treated mice compared to vehicle treated animals (Figure 3C). The number of tumor-infiltrating DCs was also significantly decreased in AZD1480 treated mice, while the maturation status of these DCs did not differ between AZD1480 treated mice compared to vehicle control treated mice (data not shown). Consistent with the observations in the spleen, we also observed a decrease in the percentage of both moMDSCs and grMDSCs within the tumor microenvironment (Figure 3D) after treatment with AZD1480. These data indicate that AZD1480 treatment has different effects on the immune cell composition of the peripheral lymphoid organs compared to the tumor microenvironment. Thus, whereas we observed an influx of T cells and a reduction of MDSC numbers in the spleen of AZD1480 treated mice, in the tumor the number of both tumor-infiltrating T cells and tumor-infiltrating MDSCs is reduced. A similar reduction was also observed for tumor-infiltrating DC numbers.

### *In vivo* AZD1480 treatment enhances the suppressive function of myeloid-derived suppressor cells

**Figure 4:**
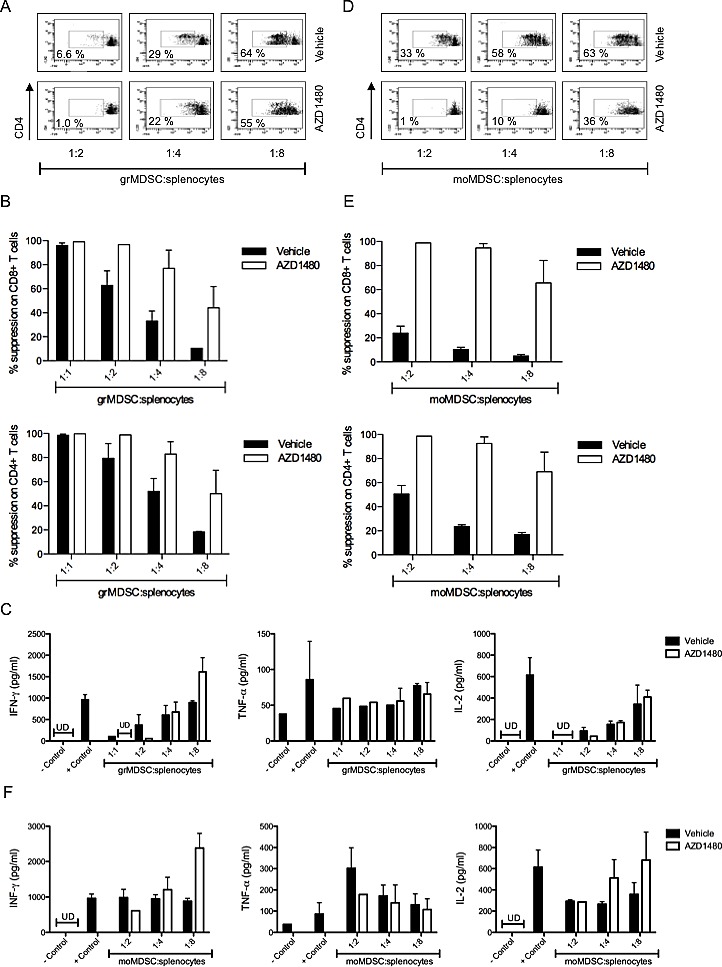
*In vivo* AZD1480 treatment negatively impacts on the suppressive function of myeloid-derived suppressor cells MO4 tumor-bearing mice were treated with AZD1480 at 30 mg/kg or vehicle control by oral gavage bid for 7 days. Two hours after the last dosing mice were sacrificed and grMDSC and moMDSC were isolated from the spleen and used in a suppression assay. Controls included T cells cultured in the absence of MDSCs with and without T-cell stimulation. A. Representative FACS profile of the proliferation of CD4^+^ T cells in the presence of different ratios of grMDSC. B. Overview of the percentage suppression of CD8^+^ T-cell proliferation (upper panel) and CD4^+^ T-cell proliferation (lower panel) cultured in the presence of different ratios of grMDSC. Two independent experiments were performed and results are presented as mean ± SEM. C. IFN-γ, TNF-α and IL-2 production by splenocytes was determined after 3 days of co-culture with different ratios of grMDSC. Results of two independent experiments are shown and data are presented as mean ± SEM. D. Representative FACS profile of the proliferation of CD4^+^ T cells in the presence of different ratios of moMDSC. E. Overview of the percentage suppression of CD8^+^ T-cell proliferation (upper panel) and CD4^+^ T-cell proliferation (lower panel) cultured in the presence of different ratios of moMDSC. Two independent experiments were performed and results are presented as mean ± SEM. F. IFN-γ, TNF-α and IL-2 production by splenocytes was determined after 3 days of co-culture with different ratios of moMDSC. Results of two independent experiments are shown and data are presented as mean SEM.

The observed reduction in the number of MDSCs in both the spleen and the tumor microenvironment prompted us to investigate whether AZD1480 affects the suppressive activity of these cells. Therefore we co-cultured splenocytes derived from healthy animals with grMDSC or moMDSC sorted from spleens of AZD1480 treated MO4 tumor-bearing mice and analyzed their effect on the proliferation of CD4^+^ and CD8^+^ T cells, respectively. At a 1:1 ratio (grMDSC:splenocytes) grMDSC isolated from vehicle treated mice and AZD1480 treated mice were equally suppressive, with a complete suppression of proliferation of both CD8^+^ and CD4^+^ T cells. For the grMDSCs isolated from vehicle treated mice we observed a dose-dependent reduction in the suppressive activity of these cells, with a complete abrogation of the suppressive function of these cells when cultured at a 1:8 (grMDSC:splenocytes) ratio. However, grMDSCs isolated from AZD1480 treated mice still yielded a 50% suppression of the proliferation of both CD4^+^ and CD8^+^ T cells when cultured at a 1:8 (grMDSC:splenocytes) ratio (Figure 4A-B). In contrast, when we looked at the cytokine secretion we observed an increase in the IFN-γ secretion when we co-culture grMDSCs isolated from the spleen of AZD1480 treated mice, compared to grMDSCs isolated from vehicle treated mice (Figure 4C). The secretion of IL-2 and TNF-α was not differentially affected by vehicle or AZD1480 treatment (Figure 4C). At a 1:2 ratio (moMDSC:splenocytes) moMDSC isolated from vehicle treated mice almost completely lost their suppressive activity on CD8^+^ T cell proliferation, while they were still capable of suppressing the proliferation of CD4^+^ T cells. However, moMDSCs isolated from AZD1480 treated mice still suppress the proliferation of both CD4^+^ and CD8^+^ T cells up to 80%, even at a 1:8 ratio (Figure 4D-E). Both the IFN-γ and the IL-2 secretion were increased when splenocytes were co-cultured in the presence of moMDSC derived from AZD1480 treated mice compared to moMDSC isolated from vehicle treated mice (Figure 4F). These data indicate that, on a per cell basis, AZD1480 enhances the suppressive function of both grMDSCs and moMDSCs on the proliferation of T cells.

### AZD1480 and ruxolitinib do not affect proliferation of T cells but negatively impact on their IFN-γ secretion *in vitro*

**Figure 5:**
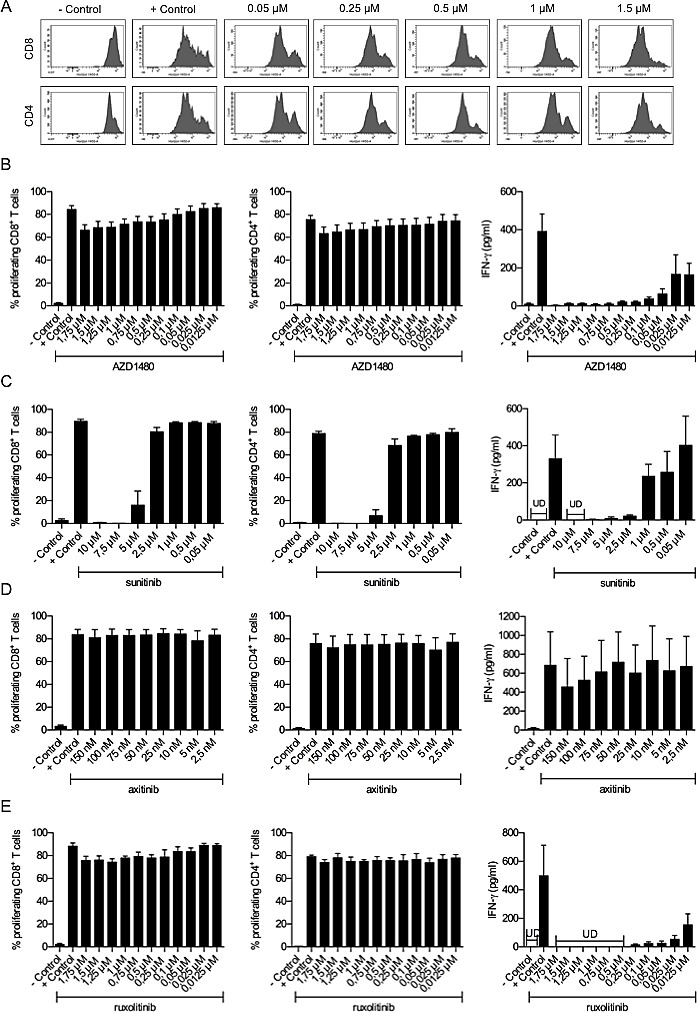
AZD1480 and ruxolitinib do not affect proliferation of murine T cells but negatively impact on their IFN-γ secretion *in vitro* Splenocytes of naive mice were treated with different concentrations of AZD1480, sunitinib, axitinib or ruxolitinib and after three days, proliferation of CD4^+^ and CD8^+^ T cells was determined by flow cytometry. Controls included T cells with and without T-cell stimulation. A. Representative FACS profile of the proliferation of CD8^+^ and CD4^+^ T cells in the presence of different concentrations of AZD1480. B. Overview of the proliferation of CD8^+^ and CD4^+^ T cells in the presence of different concentrations of AZD1480. Five independent experiments were performed and results are presented as mean ± SEM. IFN-γ secretion by splenocytes was determined after three days of culture in the presence of different concentrations of AZD1480. Five independent experiments were performed and results are presented as mean ± SEM. C. Overview of the proliferation of CD8^+^ and CD4^+^ T cells in the presence of different concentrations of sunitinib. Three independent experiments were performed and results are presented as mean ± SEM. IFN-γ secretion by splenocytes was determined after three days of culture in the presence of different concentrations of sunitinib. Three independent experiments were performed and results are presented as mean ± SEM. D. Overview of the proliferation of CD8^+^ and CD4^+^ T cells in the presence of different concentrations of axitinib. Three independent experiments were performed and results are presented as mean ± SEM. IFN-γ secretion by splenocytes was determined after three days of culture in the presence of different concentrations of axitinib. Three independent experiments were performed and results are presented as mean ± SEM. E. Overview of the proliferation of CD8^+^ and CD4^+^ T cells in the presence of different concentrations of ruxolitinib. Three independent experiments were performed and results are presented as mean ± SEM. IFN-γ secretion by splenocytes was determined after three days of culture in the presence of different concentrations of ruxolitinib. Three independent experiments were performed and results are presented as mean ± SEM.

Since we observed that AZD1480 enhanced the suppressive function of MDSCs *in vivo* we wondered whether AZD1480 had a direct effect on the proliferative capacity and cytokine secretion of T cells *in vitro*. Even at the highest concentration of AZD1480 (1.75 μM) we tested, no statistically significant difference in the proliferative capacity of either the CD4^+^ and CD8^+^ T cells was observed compared to the positive control (cells stimulated with anti-CD3/CD28 beads in the absence of AZD1480, 75 vs 63 % for the CD4^+^T cells and 83 vs 66 % for the CD8^+^T cells, respectively; Figure 5A-B). In contrast, the IFN-γ secretion of splenocytes was dramatically reduced after the addition of AZD1480, even at the lowest concentration used (0.0125 μM), compared to the control condition (Figure 5B). To rule out the possibility that off-target effects of AZD1480 mediate the observed effects on T-cell proliferation and IFN-γ secretion we performed the same experiments with ruxolitinib, a more selective inhibitor of JAK1/2, with a different off-target activity profile than AZD1480, sunitinib, which inhibits signalling through different receptor tyrosine kinases including vascular endothelial growth factor receptor (VEGFR) 1-3, platelet-derived growth factor receptor, stem cell factor receptor c-kit, flt3 and RET kinases and axitinib, a potent tyrosine kinase receptor with increased selectivity for VEGFR 1-3 and no known effect on JAK 1/2. Sunitinib treatment not only led to a dramatic reduction in the secretion of IFN-γ but also led to a dose-dependent reduction in the proliferative capacity of both CD4^+^ and CD8^+^ T cells (Figure 5C). In contrast, axitinib did not affect the proliferation of CD4^+^ and CD8^+^ T cells nor their capacity to secrete IFN-γ (Figure 5D). As was observed for AZD1480, ruxolitinib did not affect the proliferation of CD4^+^ and CD8^+^ T cells, whereas a dramatic reduction in the secretion of IFN-γ was observed (Figure 5E). Moreover, in accordance with what we observed for AZD1480, *in vitro* treatment with ruxolitinib did not induce apoptosis in MO4 melanoma tumor cells but it did inhibit P-STAT3 expression in these cells (Supplementary figure 2). These observations indicate that the observed effects of AZD1480 on T-cell proliferation and IFN-γ secretion are most likely mediated by inhibition of JAK1/2 and are not due to off-target effects of the drug.

### AZD1480 treatment does not affect the suppressive capacity of human myeloid-derived suppressor cells although it negatively impacts on the proliferation and IFN-γ secretion of human CD8^+^ T cells *in vitro*

**Figure 6:**
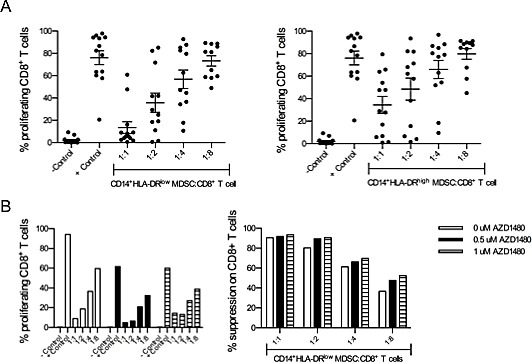
CD14HLA-DR MDSCs suppress the proliferation of CD8 T cells but AZD1480 treatment does not affect their suppressive activity Both CD14^+^HLA-DR^low^ myeloid-derived suppressor cells and CD14^+^HLA-DR^high^ monocytes were sorted from peripheral blood mononuclear cells and co-cultured for 6 days with autologous CD8^+^ T cells in the presence of anti-CD3/CD28 beads. Controls included T cells cultured in the absence of MDSCs with and without T-cell stimulation. A. Overview of the percentage of proliferating CD8^+^ T cells cultured in the presence of different ratios of myeloid-derived suppressor cells (left panel) and monocytes (right panel). B. Different concentrations of AZD1480 were added to co-cultures of myeloid-derived suppressor cells and CD8^+^ T cells and after 6 days proliferation of T cells was determined. The percentage of proliferating CD8^+^ T cells (left panel) and the percentage of suppression (right panel) are shown.

**Figure 7:**
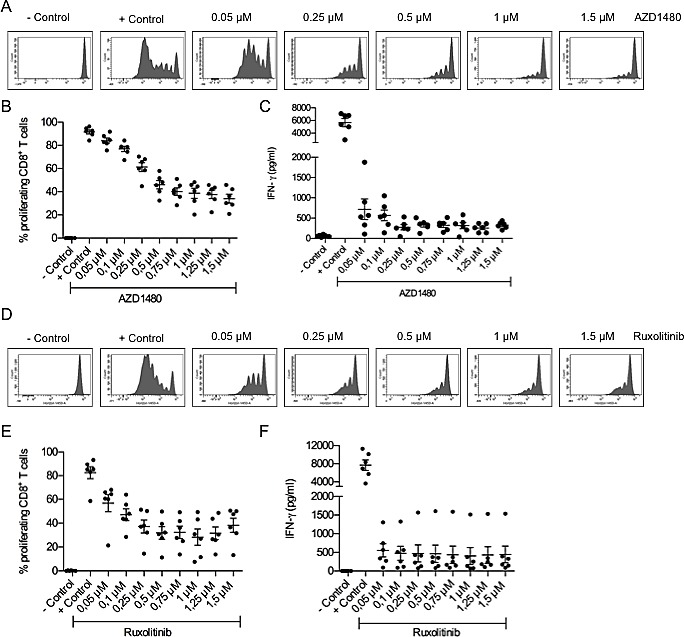
AZD1480 impairs the proliferation of human CD8 T cells and negatively impacts on their IFN-γ secretion *in vitro* CD8^+^ T cells were isolated from peripheral blood mononuclear cells obtained from melanoma patients and cultured for 6 days in the presence of anti-CD3/CD28 beads and different concentrations of AZD1480 or ruxolitinib. Controls included T cells with and without T-cell stimulation. A. Representative FACS profile of the proliferation of CD8^+^ T cells in the presence of different concentrations of AZD1480. B. Overview of the proliferation of CD8^+^ T cells in the presence of different concentrations of AZD1480. T cells of 6 different patients were analyzed and results are presented as mean ± SEM. C. IFN-γ secretion by CD8^+^ T cells cultured in the presence of different concentrations of AZD1480. T cells of 6 different patients were analyzed and results are presented as mean ± SEM. D. Representative FACS profile of the proliferation of CD8^+^ T cells in the presence of different concentrations of ruxolitinib. E. Overview of the proliferation of CD8^+^ T cells in the presence of different concentrations of ruxolitinib. T cells of 6 different patients were analyzed and results are presented as mean ± SEM. F. IFN-γ secretion by CD8^+^ T cells cultured in the presence of different concentrations of ruxolitinib. T cells of 6 different patients were analyzed and results are presented as mean ± SEM.

Although initially most studies with MDSCs were performed in mouse tumor models, there is evidence that MDSCs are also present in cancer patients[26]. A population of CD14^+^HLA-DR^low^ cells with suppressive activity on T cells has been described for several tumor types, including melanoma[27,28]. Here, we confirmed that CD14^+^HLA-DR^low^ cells, isolated from peripheral blood mononuclear cells obtained from melanoma patients, have the ability to suppress the proliferation of autologous CD8^+^ T cells in a dose-dependent manner (Figure 6A). The addition of AZD1480 to co-cultures of human MDSCs and CD8^+^ T cells did not affect the suppressive capacity of the MDSCs (Figure 6B). However, AZD1480 dramatically reduces the IFN-γ secretion by CD8^+^ T cells at very low concentrations and we observed a dose-dependent decrease in the proliferative capacity of CD8^+^ T cells treated with AZD1480 (Figure 7A-C). To exclude possible off-target effects of AZD1480, CD8^+^ T cells were treated with ruxolitinib. When used at the same concentrations as AZD1480, ruxolitinib has the capacity to reduce the proliferation of CD8^+^ T cells and to decrease their ability to secrete IFN-γ (Figure 7D-F).

## DISCUSSION

We have shown that, although AZD1480 has the ability to delay tumor growth and prolong survival of MO4 tumor-bearing mice, it has a significant impact on several aspects of the immune system. The prolonged survival in response to AZD1480 is quite mild and may be associated with the detrimental effects on the immune system. Thus, AZD1480 impairs the proliferative as well as the IFN-γ secretion capacity of T cells while at the same time enhancing the suppressive capacity of MDSCs. The same effects were observed with ruxolitinib, a more specific JAK1/2 inhibitor that has been approved by the FDA for patients with intermediate- to high-risk myelofibrosis[29]. Moreover, sunitinib, which inhibits signalling through different receptor tyrosine kinases including vascular endothelial growth factor receptor (VEGFR) 1-3, platelet-derived growth factor receptor, stem cell factor receptor c-kit, flt3 and RET kinases, also reduced the capacity of murine CD4^+^ and CD8^+^ T cells to secrete IFN-γ, while axitinib, a potent tyrosine kinase receptor with increased selectivity for VEGFR 1-3, and no known activity for JAK1/2, did not influence the proliferation of T cells nor their capacity to secrete IFN-γ. These results indicate that the observed immunological effects are mediated by the inhibition of the JAK-STAT pathway.

JAK 1 and 2 are ubiquitously expressed in mammals where they are involved in cell growth, survival, development and differentiation of immune cells[30,31]. Complete loss of function of either JAK1 or JAK2 is lethal in mice and has not been described in humans[32,33]. However, activating mutations of JAKs are found in association with malignant transformations, the most common being the gain-of-function V617F mutation of JAK2 in polycythemia vera and other myeloproliferative disorders[34]. In solid cancers JAK gene mutations are rare events[35,36]. Despite this, we show that the JAK1/2 inhibitor, AZD1480, is able to inhibit tumor growth and prolong survival in a murine melanoma model. This is in line with observations of Hedvat *et al*. who showed that AZD1480 was able to suppress the growth of solid tumor xenograft cell lines that displayed constitutive STAT3 activation[22]. However, Hedvat *et al*. performed their experiments in athymic nude mice so they were unable to evaluate the role of the immune system in the observed tumor regression.

Further research is needed to fully unravel the mechanism used by AZD1480 to delay tumor growth while at the same time inhibiting T-cell effector functions. Interestingly, similar effects have been described for mTOR inhibitors, the so-called rapalogs. Thus, it has been shown for different rapalogs, including rapamycin, that in addition to their potent immunosuppressive function, they can also act as cancer-preventive agents. Both actions are mediated through specific inhibition of the mTOR signaling pathway. It has been shown that the mTOR pathway plays an important role in the regulation of the cell cycle in response to changes in nutrient levels [37,38]. Moreover, the phosphatidylinositol 3-kinase (PI3K)/mTOR pathway is constitutively activated in different types of cancer and involves a number of tumor suppressor genes including PTEN, LKB1, TSC1, TSC2, Rb1 and p53, making this pathway a promising target for anti-cancer therapies[39–41]. Rapamycin inhibits tumor growth by affecting tumor cell proliferation, inducing apoptosis and by suppressing angiogenesis. However the same mechanisms are used to inhibit the proliferation of B and T cells, resulting in immunosuppression. The available evidence indicates that the anti-cancer activities may be dominant over the immunosuppressive effects. The same could be true for AZD1480, and other JAK inhibitors. It has for example been shown that AZD1480 has anti-angiogenic effects by itself[23] and that AZD1480 improves the effect of cediranib, a potent VEGFR kinase inhibitor, in tumors refractory to anti-VEGFR treatment[42]. It is possible that inhibition of the JAK/STAT pathway could indirectly influence the activation of mTOR, thereby suppressing tumor growth[43]. However further research is needed to confirm this hypothesis and to elucidate the exact working mechanisms of AZD1480.

Despite the inhibition of tumor growth *in vivo, in vitro* treatment with AZD1480 did not induce apoptosis in either mouse or human melanoma cell lines. One possibility for this discrepancy is that JAK/STAT signaling is not required for cell survival in cell culture in which the cells are exposed to an excess of growth factors present in the serum. In the *in vivo* situation, the complexity of the tumor environment could provide a context in which JAK/STAT activity is essential for survival. Although it has been shown that AZD1480 suppresses P-STAT3 expression both in the tumor cells as well as in the different types of cells present in the tumor microenvironment, the relative contribution of the different cell types to the observed *in vivo* effects is not clear at this moment.

STAT3 is probably one of the most important transcription factors that regulate MDSC expansion and function. It has been shown that ablation of STAT3 expression markedly reduced the expansion of MDSCs and promoted the accumulation of DCs in tumor-bearing mice[12,13]. We indeed show that treatment of MO4 tumor-bearing mice with AZD1480 leads to a reduction in the number of MDSCs both in the spleen and in the tumor microenvironment. However, we did not observe differences in the number of DCs nor in the maturation status of these DCs. This is in line with observations made by Heine *et al*. who showed that the JAK-inhibitor ruxolitinib affects DC differentiation, phenotype and function leading to impaired T-cell activation[44]. This explains the use of this inhibitor for the treatment of autoimmune diseases[45,46]. Hipp *et al*. showed that sorafenib, but not sunitinib, inhibits the function of DCs and the induction of primary immune responses[47]. Moreover, we showed that although treatment with AZD1480 leads to a reduction in the number of MDSCs, the suppressive activity of these MDSCs is enhanced compared to the MDSCs obtained from vehicle treated mice. Indicating that STAT3 does play an important role in the accumulation of these MDSCs while the role of STAT3 in the function of these cells still remains unclear. In tumor-bearing mice two functionally distinct subtypes of MDSCs have been identified: CD11b^+^Ly6G^+^Ly6C^low^ MDSCs which are morphologically similar to polymorphonuclear granulocytes (grMDSCs) and CD11b^+^Ly6G^−^Ly6C^high^ MDSCs which have a monocytic phenotype (moMDSCs). We have previously shown that grMDSCs possess a stronger suppressive activity compared to moMDSCs. Moreover, both grMDSCs and moMDSCs isolated from within the tumor microenvironment have a much stronger suppressive activity compared to MDSCs isolated from the spleen of tumor-bearing mice, associated with a higher NO_2_^−^ production by the tumor-derived moMDSCs and a higher arginase activity for both subsets[48]. Since it has been shown that the expression of P-STAT3 regulates arginase 1 expression levels and activity in MDSCs[49], and since we observed a higher activity of arginase 1 in tumor-derived MDSCs, we cannot exclude that AZD1480 has differential effects on the function of either tumor-or spleen-derived MDSCs. In favor of this hypothesis are the results of Ko *et al.*, who showed that sunitinib not only has divergent effects on the subpopulations of MDSCs but also differentially impacts on the suppressive function of spleen-and tumor-derived MDSCs[50]. However, given the difficulty to obtain MDSCs from a solid tumor we were not able to test the differential effects of AZD1480 on subsets of spleen-and tumor-derived MDSCs.

In contrast to the murine data, addition of AZD1480 to co-cultures of MDSCs and T cells obtained from melanoma patients did not affect the suppressive activity of MDSCs. The difference between the murine and human data could be explained by the fact that mice were treated *in vivo*, while in the human scenario the drug was added *in vitro*. This could indicate that AZD1480 does not have a direct effect on the MDSCs but that this drug affects the tumor microenvironment leading to alterations in the cytokine secretion.

Both the murine and human T-cell function is affected by the addition of either AZD1480 or ruxolitinib to *in vitro* cultures. This is in line with observations made by Liu *et al*. who showed that *in vivo* treatment with AZD1480 leads to a reduced proliferation and cytokine secretion of T cells in a mouse model of experimental autoimmune encephalomyelitis[51]. In contrast, Heine *et al*., showed that ruxolitinib predominantly works on the function of DCs and not on T cells[44]. However, differences between *in vitro* and *in vivo* treatment could explain these contradictory results. In a recent paper, Miller *et al*. show that increased JAK2 expression in primary breast tumors is associated with increased intratumoral lymphocyte infiltration, as well as improved clinical outcome. Moreover, they confirmed our finding that ruxolitinib dramatically inhibits IFN-γ production by T cells *in vitro*[52].

Taken together, caution should be taken when treating cancer patients with JAK-STAT inhibitors, especially when combining them with immunotherapy, since systemic targeting of the JAK-STAT pathway can have divergent effects on tumor growth and anti-tumor immune responses.

Currently, novel inhibitors with a higher selectivity for either JAK1 or JAK2 are being developed[53]. Future immunological studies should address whether the immunosuppressive effects are also observed with these selective inhibitors, especially when considering their use for the treatment of cancer patients in combination with active immunotherapy.

## MATERIAL AND METHODS

### Tumor Cell lines

The mouse melanoma cell line MO4 (kindly provided by K. Rock, University of Massachusetts Medical center) was cultured in Roswell Park Memorial Institute (RPMI)-1640 medium (Sigma) supplemented with 5% fetal clone I (FCI), 100 U/ml penicillin, 100 μg/ml streptomycin, 2 mM L-glutamine, 1 mM sodium pyruvate and non-essential amino acids. For some experiments MO4 cells were treated with IL-6 (50 ng/ml, Gentaur) in order to upregulate P-STAT3 expression. The K1735-C4 melanoma cell line (kindly provided by I.J. Fidler, University of Texas) is maintained in Dulbecco's Modified Eagle Medium (DMEM, Sigma) supplemented with 10% fetal bovine serum (FBS), 2mM L-glutamine, 100 U/ml penicillin and 100 μg/ml streptomycin. The human melanoma cell line 1087-mel (provided by S.L. Topalian, Sidney Kimmel Comprehensive Cancer Center, Johns Hopkins University) was cultured in RPMI-1640 medium supplemented with 10% FBS, 100 U/ml penicillin, 100 μg/ml streptomycin, 2 mM L-glutamine, 1 mM sodium pyruvate and non-essential amino acids. No full authentication of the cell lines was carried out. Cell lines were tested for their known characteristics including expression of antigens and MHC molecules by reverse transcriptase PCR or flow cytometry. Their *in vitro* and *in vivo* growth characteristics were closely monitored.

### Mice and tumor model

Female, 6- to 12-week-old C57BL/6 mice were purchased from Charles River (L'Arbresle, France). Animals were treated according to the European guidelines for animal experimentation. All experiments were reviewed and approved by the ethical committee for use of laboratory animals of the Vrije Universiteit Brussel. For the induction of tumor growth, mice were anesthetized by inhalation of isoflurane and inoculated with 5 × 10^5^ MO4 tumor cells administered by subcutaneous injection in the lower back.

### Preparation of AZD1480, sunitinib, axitinib and ruxolitinib

AZD1480 was kindly provided by Dr. Dennis Huszar from Astrazeneca. Ruxolitinib, axitinib and sunitinib were obtained from Selleckchem. For *in vitro* experiments all drugs were dissolved in 100% DMSO at a final stock concentration of 10 mM and stored, in single-use vials, at -20°C. For *in vivo* experiments AZD1480 was formulated in sterile water supplemented with 0.5% hydroxy-propyl-methylcellulose (Sigma) and 0.1% Tween-80 (Sigma).

### Treatment of tumor-bearing mice with AZD1480

Ten days after the inoculation of MO4 tumor cells, when tumors reached a diameter of approximately 100 mm^3^, mice were randomly divided into a control group and a treatment group (5 mice/group) which were dosed orally with vehicle or AZD1480 (30 mg/kg), respectively. Mice were treated by oral gavage twice a day (bid) for a period of 7 days. At this dose of AZD1480 no lethal toxicity was observed. Tumors were measured every 2 days and tumor volume was calculated based on the following formula: 0.5 x ((smallest diameter)^2^ x (largest diameter)). When tumors reached a volume of 2500 mm^3^ mice were sacrificied.

### *In vitro* apoptosis assay

MO4, K1735-C4 and 1087-mel cells were seeded in 6-well plates (0.5 ×10^6^ cells/well) overnight to allow cell adhesion. Cells were subsequently treated with different concentrations of AZD1480 (0.5 μM, 1 μM, 5 μM and 10 μM) and 48 hours later, the percentage of apoptotic cells was determined by staining with Annexin-V Alexa Fluor 647 (AF647, BioLegend) and 7-AAD (BD Biosciences).

### Preparation of a single cell suspension from spleen and tumor of tumor-bearing mice

For some experiments tumor-bearing mice were sacrificed 2 hours after the last treatment with AZD1480 or vehicle and spleens and tumors were isolated. Single-cell suspensions prepared from splenocytes were treated with Tris-buffered ammonium chloride for 5 min to remove red blood cells. Single-cell suspensions from tumor tissue were prepared using the GentleMACS single cell isolation protocol (Miltenyi Biotec). Briefly, tumors were isolated and minced into small pieces followed by a mechanical dissociation step using the GentleMACS dissociator. Samples were then incubated for 40 min at 37°C with the following enzymes: collagenase I (10,000 U/ml) and dispase II (32 mg/ml). After a last mechanical disruption step, the digested tumors were harvested, filtered (over a 70 μM nylon filter, BD Falcon) and red blood cells were lysed by adding Tris-buffered ammonium chloride.

### Western blot analysis

Cells were lysed with RIPA buffer (150mM NACl, 1% NP-40, 50 mM Tris, 1 mM EDTA, 1 mM Na_3_VO_4_) supplemented with 1x protease inhibitors (Roche). The total amount of protein in the lysates was quantified using the Pierce^TM^ BCA Protein Assay Kit, according to the manufacturer's instructions. Proteins (10 μg) were separated on a 12% SDS-PAGE and subsequently transferred to a nitrocellulose membrane. A monoclonal mouse antibody against P-STAT3 was used as primary antibody and a horseradish peroxidase (HRP)-conjugated anti-mouse immunoglobulin G (IgG) antibody was used for detection. An HRP-conjugated β-actin antibody was used for normalization of the signal. All antibodies were obtained from Cell Signaling. Antibody binding was visualized with enhanced chemoluminescence (Pierce).

### Phenotypical characterization of immune cells

In order to evaluate the phenotype of different immune cell populations, cells derived from the spleen or tumor of vehicle- or AZD1480-treated mice were stained with the following antibodies: phycoerythrin (PE)-Cy7-conjugated anti-mouse CD3 (BioLegend), Alexa Fluor 700 (AF700)-conjugated anti-mouse CD4 (BD Biosciences), AF647-conjugated anti-mouse CD8 (BioLegend), Horizon V450-conjugated anti-mouse CD45 (BD Biosciences), peridinin chlorophyll protein (PerCP)-Cy5.5-conjugated anti-mouse CD4 (BD Biosciences), PE-conjugated anti-mouse CD25 (eBioscience), AF700-conjugated anti-mouse CD127 (eBioscience), AF647-conjugated anti-mouse CD11c (BioLegend), PE-conjugated anti-mouse CD11b (BD Biosciences), fluorescein isothiocyanate (FITC)-conjugated anti-mouse CD86 (BD Biosciences), allophycocyanin (APC)-H7-conjugated anti-mouse CD80 (BD Biosciences), FITC-conjugated anti-mouse CD11b (BD Biosciences), AF647-conjugated anti-mouse Ly6G (BioLegend) and PECy7-conjugated anti-mouse Ly6C (BioLegend).

### Purification of MDSCs from spleen

Different subsets of MDSCs were purified from the spleen of tumor-bearing mice as described before[48]. Briefly, the CD11b^+^ cell fraction was enriched by MACS sorting using CD11b MicroBeads (Miltenyi Biotec) according to the manufacturer's instructions. These enriched CD11b^+^ cells were then stained with FITC-conjugated anti-mouse CD11b, APC-conjugated anti-mouse Ly6G and PECy7-conjugated anti-mouse Ly6C antibodies. Subsets of MDSCs were sorted to a purity of > 90% using a BD FACSAria III cell sorter (BD Biosciences).

### Isolation of MDSCs and T cells from the blood of melanoma patients

The leukapheresis products of melanoma patients participating in TriMix-DC-based immunotherapy trials (NCT01066390) were elutriated (Elutra Cell Seperation System; Caridian BCT) using a previously described technique[54]. Fractions 2 and 3 were used as a source for T cells, whereas fraction 5, containing monocytes, was used for the isolation of MDSCs. First, the CD14^+^ cell fraction was enriched by MACS sorting using CD14 MicroBeads (Miltenyi Biotec) according to the manufacturer's instructions. These enriched CD14^+^ cells were then stained with APC-Cy7-conjugated anti-human CD14 (BioLegend) and a PE-conjugated anti-human HLA-DR (BD Biosciences). CD14^+^HLA-DR^high^ cells (considered to be monocytes) and the CD14^+^HLA-DR^low^ cells (defined as MDSCs) were sorted to a purity of > 90% using a BD FACSAria III cell sorter (BD Biosciences). Lymphocytes were purified from fractions 2 and 3 of the elutriation product and were used as a source of T cells. CD8^+^ T_eff_ cells and naïve CD4^+^ T cells were sorted on LS columns using MACS CD8^+^ or CD4^+^ magnetic beads, respectively (Miltenyi Biotec).

### Suppression assay

The suppressive activity of MDSCs was determined using standard proliferation assays as described before[47]. In brief, freshly isolated splenocytes, obtained from healthy mice, were labelled with 0.5 μM CellTrace^TM^ Violet (Invitrogen) and seeded in 96-well plates at 2 × 10^5^ cells/well. Purified MDSCs were then added at different ratios, ranging from 1:1 to 1:8 (MDSC:splenocytes). T-cell proliferation was induced by anti-CD3/CD28 beads (Invitrogen) in the presence of IL-2 (100 U/ml, Chiron). After 3 days, proliferation of CD4^+^ and CD8^+^ T cells was analyzed by flow cytometry by staining with PerCPCy5.5-conjugated anti-mouse CD3 (BioLegend), AF700-conjugated anti-mouse CD4 and APC-H7-conjugated anti-mouse CD8 (BD Biosciences) antibodies. The percentage of T-cell suppression was calculated using the following formula:

$$\%\text{Suppression}=\left[1-\frac{\%\text{proliferation with MDSCs}}{\%\text{proliferation without MDSCs}}\right]*100$$

In order to analyze the suppressive function of human MDSCs, allogeneic purified CD8^+^ T cells were labelled with 0.5 μM CellTrace^TM^ Violet (Invitrogen) and seeded in 96-well plates at 2 × 10^5^ cells/well. Purified CD14^+^HLA-DR^high^ monocytes and CD14^+^HLA-DR^low^ MDSCs were added at different ratios, ranging from 1:1 to 1:8 (MDSC:T cells). After 6 days, proliferation of CD8^+^ T cells was analyzed by flow cytometry by staining with APC-conjugated anti-human CD3 and FITC-conjugated anti-human CD8.

To determine the direct effects of AZD1480 and ruxolitinib on the proliferation of both murine and human T cells, 2 × 10^5^ CellTrace^TM^ Violet labelled T cells were seeded in 96-well plates and different concentrations of AZD1480 or ruxolitinib were added (ranging from 0-1.75 μM). Proliferation of CD8^+^ T cells was analyzed by flow cytometry as described before.

### Flow cytometry

Data were collected using an LSR Fortessa flow cytometer (BD Biosciences) and analyzed with FACSDiva (BD Biosciences) software.

### ELISA

The concentration of interferon-gamma (IFN-γ), tumor-necrosis factor-alpha (TNF-α) and interleukin-2 (IL-2) in culture supernatants was quantified using commercially available ELISA kits (all from eBioscience) according to the manufacturer's instructions. The optical density was measured at 450 nm using a Thermomax microplate reader.

### Statistical analysis

Results are presented as mean SEM. For the comparison of two groups, unpaired Student's t-test was carried out. Sample size and number of repetitions for each experiment are indicated in the figure legends. Survival was visualized in a Kaplan-Meier curve and analyzed by the log-rank test. All statistical analyses were performed using GraphPad Prism 5.

## SUPPLEMENTARY MATERIAL FIGURES


