# Supplementary material for: AZD1480 delays tumor growth in a melanoma model while enhancing the suppressive activity of myeloid-derived suppressor cells
Source: Oncotarget. 2014 Jul 25;5(16):6801–15. doi: 10.18632/oncotarget.2254 (PMC4196164; doi:10.18632/oncotarget.2254)
Supplement: Supplementary file 1 [file oncotarget-05-6801-s001.pdf]

AZD1480 delays tumor growth in a melanoma model while enhancing the suppressive activity of myeloid-derived suppressor cells

Supplementary Material

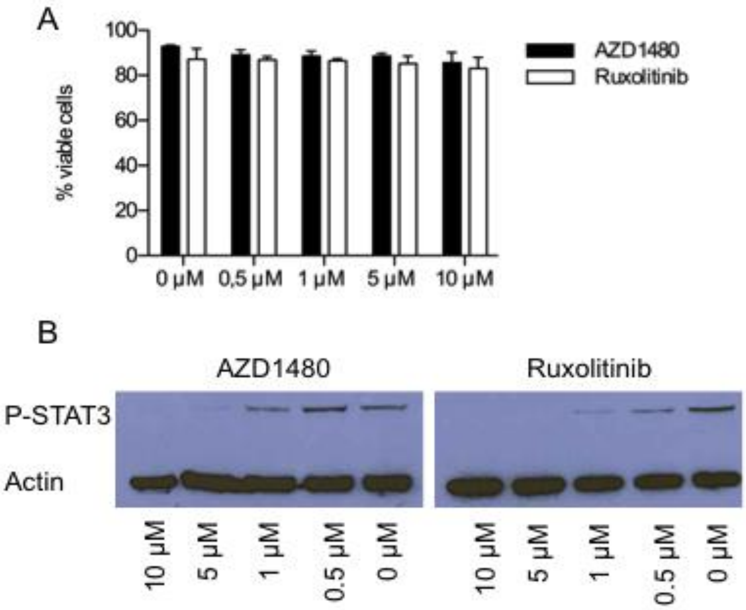

Supplementary Figure 1: AZD1480 and ruxolitinib do not induce apoptosis in a human melanoma cell line but do inhibit P-STAT3 expression in vitro. 1087-mel melanoma cells were treated with the indicated doses of either AZD1480 or ruxolitinib and after 48 hours the percentage of apoptotic cells was determined by flow cytometry using Annexin-V/7-AAD staining. A. Overview of the percentage of viable cells (defined as Annexin-V$^-$/7-AAD$^-$ cells) in the 1087-mel melanoma cell line 48 hours after the addition of different concentrations of AZD1480 or ruxolitinib. Results of 3 independent experiments are shown as mean ± SEM. B. 1087-mel cells were treated with the indicated doses of AZD1480 or ruxolitinib and after 6 hours the level of P-STAT3 expression was determined by western blotting. One representative blot of 2 independent experiments is shown.

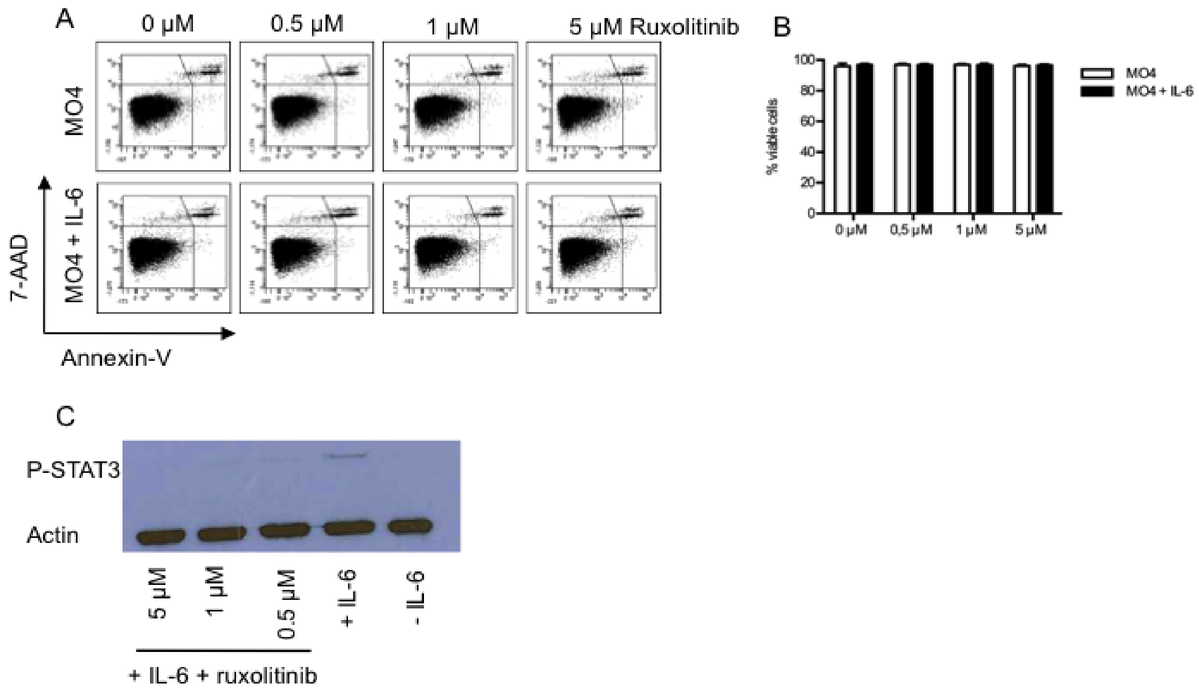

Supplementary Figure 2: Ruxolitinib inhibits P-STAT3 expression but does not induce apoptosis in the MO4 melanoma cell line in vitro. MO4 cells and MO4 cells pretreated with IL-6 (50 ng/ml) were treated with the indicated doses of ruxolitinib and after 48 hours the percentage of apoptotic cells was determined by flow cytometry using Annexin-V/7-AAD staining. **A.** Representative FACS profile of Annexin-V/7-AAD staining of MO4 cells treated with ruxolitinib. **B.** Overview of the percentage of viable cells (defined as Annexin-V$^-$/7-AAD$^-$ cells) in the MO4 melanoma cell line 48 hours after the addition of different concentrations of ruxolitinib. Results of 3 independent experiments are shown as mean ± SEM. **C.** MO4 cells pretreated with IL-6 (50 ng/ml) were treated with the indicated doses of ruxolitinib and after 6 hours the level of P-STAT3 expression was determined by western blotting. One representative blot of 2 independent experiments is shown.
